# Risk factors and outcomes associated with postoperative ileus following ileostomy formation: a retrospective study

**DOI:** 10.1186/s13741-021-00226-z

**Published:** 2021-12-13

**Authors:** Anya L. Greenberg, Yvonne M. Kelly, Rachel E. McKay, Madhulika G. Varma, Ankit Sarin

**Affiliations:** 1 School of Medicine, University of California, San Francisco, 513 Parnassus Ave #S-245, San Francisco, CA 94143 USA; 2 Department of Surgery, University of California, San Francisco, 513 Parnassus Ave #S-321, San Francisco, CA 94143 USA; 3 Department of Anesthesia and Perioperative Care, University of California, San Francisco, San Francisco, CA USA; 4 Department of Surgery, University of California, San Francisco, 550 16th Street, San Francisco, CA 94158 USA

**Keywords:** Postoperative ileus, Ileostomy formation, Fluid balance

## Abstract

**Background:**

Postoperative ileus (POI) is associated with increased patient discomfort, length of stay (LOS), and healthcare cost. There is a paucity of literature examining POI in patients who have an ileostomy formed at the time of surgery. We aimed to identify risk factors for and outcomes associated with POI following ileostomy formation.

**Methods:**

We included 261 consecutive non-emergent cases that included formation of an ileostomy by a board-certified colorectal surgeon at our institution from July 1, 2015, to June 30, 2020. Demographic, clinical, and intraoperative factors associated with increased odds of POI were evaluated. Post-procedure LOS, hospitalization cost, and re-admissions between patients with and without POI were compared.

**Results:**

Out of 261 cases, 85 (32.6%) were associated with POI. Patients with POI had significantly higher body mass index (BMI) than those without POI (26.6 kg/m^2^ vs. 24.8kg/m^2^; *p* = 0.01). Intraoperatively, patients with POI had significantly longer procedure duration than those without POI (313 min vs. 279 min; *p* = 0.02). Patients with POI had a significantly higher net fluid balance at postoperative day (POD) 2 than those without POI (+ 2.65 L vs. + 1.80 L; *p* = 0.004), with POD2 fluid balance greater than + 807 mL (determined as the maximum Youden index for sensitivity over 80%) associated with a higher rate of POI (*p* = 0.006). This difference remained significant when adjusted for age, gender, BMI, pre-operative opioid use, procedure duration, and operative approach (*p* = 0.01). Patients with POI had significantly longer LOS (11.40 days vs. 5.12 days; *p* < 0.001) and direct cost of hospitalization ($38K vs. $22K; *p* < 0.001).

**Conclusions:**

Minimizing fluid overload, particularly in the first 48 h after surgery, may be a strategy to reduce POI in patients undergoing ileostomy formation, and thus decrease postoperative LOS and hospitalization cost. Fluid restriction, diuresis, and changes in diet advancement or early stoma intubation should be considered measures that may improve outcomes and should be studied more intensively.

## Background

Postoperative ileus (POI) after abdominal colorectal surgery is associated with increased patient discomfort, hospital length of stay (LOS), and cost of care (Peters et al., 2020). Minimally invasive surgery and the elimination of opioid use are widely accepted, evidence-based measures to reduce POI (Chapman et al., 2018). Other common interventions, albeit inconsistently supported by evidence, include intraoperative fluid restriction, early diet advancement, early ambulation, and chewing gum (Chapman et al., 2018; Lobo et al., 2002; Grass et al., 2019). Enhanced Recovery after Surgery (ERAS) pathways for abdominal colorectal surgery that incorporate these measures have become widespread among surgical practices nationally. However, despite these strategies, POI is a prevalent and costly condition, affecting up to 30% of patients undergoing abdominal surgery (Venara et al., 2016) and constituting one of the most prominent factors associated with increased LOS and cost of hospitalization among the colorectal surgery population (Ahmed Ali et al., 2014; Iyer et al., 2009; Asgeirsson et al., 2010; Mao et al., 2019). Although the concept of optimal fluid balance to minimize gastrointestinal edema is broadly understood to be an important factor in preventing POI, a single best-practice strategy has not been established (Gupta & Gan, 2016).

Ileostomy formation has several indications (Rajaretnam & Lieske, 2020). It is common among patients with cancer or inflammatory bowel disease undergoing colorectal surgery (Chan et al., 2019) and is associated with an increased risk for POI. In patients undergoing anterior rectal resection, the odds of POI are up to five times higher with ileostomy formation compared to patients without an ileostomy (Reichert et al., 2018), highlighting the importance of addressing this postoperative complication in this particular cohort of patients. There is a paucity of literature examining POI in patients who have an ileostomy formed at the time of surgery. We sought to address this gap by identifying factors and outcomes associated with POI in patients undergoing formation of an ileostomy as a part of their colorectal surgery at our institution.

## Materials and methods

This study was approved by the Institutional Review Board at the University of California, San Francisco (UCSF): Study Number 18-26677. An Enhanced Recovery after Surgery (ERAS) pathway, developed in partnership with anesthesiologists, nursing staff, and other colleagues, was implemented at our institution for all patients undergoing abdominal colorectal surgery in 2014. The pathway includes the use of a multimodal pain regimen, early ambulation, and diet advancement to solids on postoperative day (POD) 1. We performed a retrospective review of all consecutive patients who had elective (dichotomized as non-urgent or urgent) abdominal colorectal surgery that included formation of an ileostomy by a board-certified colorectal surgeon at the UCSF Mission Bay or Moffitt-Long Hospital from July 1, 2015, to June 30, 2020. Patient data were obtained from an automated ERAS report from our electronic medical record (EMR) as well as our National Surgical Quality Improvement Program reports from this time period. No participants had missing data for variables of interest.

At our institution, ileostomy formation is standard in several types of procedures (Table 1). Intraoperatively, while there is no institutional standard basic fluid infusion rate, our ERAS protocol outlines a goal that fluid (crystalloid) administration should not exceed 2 L unless estimated blood loss is greater than 300 mL. Outside of this guideline, fluid is administered based on the clinical judgment of the anesthesiologist in the context of a variety of factors including estimated fluid deficits. Moreover, obligate fluid administration occurs during delivery of intravenous medications, including propofol and adjuncts such as lidocaine, magnesium, and ketamine that are encouraged in the interests of opioid sparing in the ERAS pathway. Dynamic parameters of preload (e.g., pulse contour analysis) are inconsistently employed at our institution, and no universal standard for choosing fluid bolus vs. vasopressor support for treatment of hypotension, outside of clinically obvious paradigms such as hemorrhage, currently exists. Postoperatively, fluids are typically run at 75 mL/h for patients within the normal body mass index (BMI) range of 18.5–24.9 (and higher if BMI is above the normal range) and discontinued when patients are able to tolerate a clear liquid diet, typically on the morning of POD 1. Postoperative patients are given 500-mL boluses if urine output is below 0.5 mL/kg/h, in accordance with the standard practice.

**Table 1:** Procedures in which ileostomy formation is standard at our institution

Procedure	Standard practice at our institution
Low anterior resection (any anastomosis below 8 cm from the anal verge with radiation or below 5 cm without radiation)	Diverting loop ileostomy
Total colectomy without ileorectal anastomosis	End ileostomy
Proctocolectomy without ileal pouch-anal anastomosis (“J-pouch”)	End ileostomy
Proctocolectomy with J-pouch	Diverting loop ileostomy
Completion proctectomy with J-pouch (if prior total colectomy)	Diverting loop ileostomy
Any procedure with elevated risk of anastomotic leak (e.g., prior pelvic irradiation, steroid use, etc.)	Diverting loop ileostomy

During the patient’s hospitalization, onset of POI was defined clinically by the primary team based on the findings of postoperative nausea and vomiting and/or abdominal distension and treated by restricting diet, placing of a nasogastric (NG) tube, and/or intubating the ileostomy with a red rubber Robinson catheter.

For the study, POIs were determined by reviewing each patient’s chart to identify documentation of ileus or, in the absence of explicit documentation of POI in a patient’s chart, evidence of ileus-specific intervention such as prolonged NPO status (POD3 or longer), postoperative NG tube placement, or stoma intubation with a red rubber Robinson catheter.

We used specific demographic data (age, gender, smoking history), clinical characteristics (BMI, diabetes history, preoperative steroid use, and chronic opioid use), intraoperative factors (epidural placements, procedure duration, and approach), and postoperative factors (early postoperative fluid balance [which includes all intraoperative and postoperative intake and output], postoperative opioid use) in our models to compare patients with and without POI. Since postoperative fluid balance is a continuous variable, maximum Youden’s index for sensitivity over 80% was used to obtain the optimal cutoff value that provides the best tradeoff between sensitivity and specificity (sensitivity plus specificity minus one). Based on this approach, a postoperative day (POD) 2 net fluid balance cutoff point of 807 mL was used in our analysis as a categorical variable in addition to evaluating postoperative fluid balance continuously. To assess for potential selection bias, Pearson’s correlation coefficient was used to examine the correlation between length of procedure and POD1/POD2 net fluid balance. Bivariate analyses using *t*-tests for continuous variables and Fisher’s exact tests for categorical variables were performed. Multivariable logistic regression model, including age, gender, preoperative opioid use, and operative approach, as well as variables that were statistically significant on bivariate analysis, was also performed. Finally, we compared differences in outcomes (LOS, direct cost of hospitalization, and 30-day readmission rates) between patients with new ileostomies who developed an ileus versus those who did not.

Statistical analysis was performed using SAS V9.4 (SAS Institute, Cary, NC, USA). Differences were considered statistically significant at *p* value ≤ 0.05.

## Results

A planned ileostomy was created by a colorectal surgeon in 261 elective and urgent procedures (mean age 50, 54% male) at our institution between July 1, 2015, and June 30, 2020 (Table 2). Diverting loop ileostomy (DLI) vs. end ileostomy was created for patients with an indication of ileostomy in accordance with standard practice at our institution and clinical judgment (Table 3). For patients with low anterior resections (LARs), our standard practice is to form a DLI for anastomoses at or below 5 cm from the anal verge (or below 8 cm from the anal verge if the patient had prior radiation therapy). Of 294 total LARs performed during the study period, 134 (46%) got DLI. Of all patients for whom ileostomy was created, 85 (32.6%) met our definition for POI.

**Table 2:** Demographic and clinical characteristics of study population

	All	No POI	POI	% POI	*p*-value
Absolute prevalence	261 (100%)	176	85	32.6%	–
**Demographic characteristics**					
Gender					
Female gender	119 (45.6%)	85	34	28.6%	0.23
Male gender	142 (54.4%)	91	51	35.9%	
Age (years)	50.4	49.3	52.7	–	0.092
BMI (kg/m^2^)	25.4	24.8	26.6	–	0.01
Smoking status					
Yes	7 (2.7%)	3	4	57.1%	0.22
No	254 (97.3%)	173	81	31.9%	
**Clinical characteristics**					
Diabetes					
Yes	10 (3.8%)	6	4	40.0%	0.73
No	251 (96.2%)	170	81	32.3%	
Chronic steroid use prior to surgery					
Yes	66 (25.3%)	44	22	33.3%	0.88
No	195 (74.7%)	132	63	32.3%	
Chronic opioid use prior to surgery					
Yes	58 (22.2%)	35	23	39.7%	0.21
No	203 (77.8%)	141	62	30.5%	
Indication for surgery					
Cancer	140 (53.6%)	94	46	32.9%	0.37
Inflammatory bowel disease	96 (36.8%)	68	28	29.2%	
Another condition	25 (9.6%)	14	11	44.0%	
Case type					
Elective (non-urgent)	234 (89.7%)	161	73	31.2%	0.19
Elective (urgent)	27 (10.3%)	15	12	44.4%	

*p*-values from Fisher’s exact tests for categorical variables and *t*-tests for continuous variables

*POI* postoperative ileus

**Table 3:** Ileostomy type by procedure of study population

	Ileostomy type		All
	DLI	End	
Low anterior resection	134	0	134
Total colectomy	0	54	54
Total proctocolectomy^a^	1	32	33
Total proctocolectomy with J-pouch	20	0	20
Proctectomy with J-pouch	18	0	18
Right hemicolectomy	0	2	2
**Grand total**	**173**	**88**	**261**

^a^One patient had total proctocolectomy with end ileostomy planned, but because the terminal ileum was tethered by mesenteric tumor, an ileostomy with loop configuration was created and the efferent end was stapled off to ensure bowel decompression

*DLI* diverting loop ileostomy; *End* end ileostomy; *J-pouch* ileal pouch-anal anastomosis

In our bivariate analysis, statistically significant differences were not observed in terms of age and gender when comparing the POI and non-POI groups. Preoperative clinical factors such as history of diabetes mellitus, preoperative steroid use, chronic opioid use, smoking status, urgency of surgery, and indication for surgery were also not statistically significant between the two groups. However, a statistically significant difference was observed in BMI between the two groups (26.6 kg/m^2^ [25.4 kg/m^2^–27.8 kg/m^2^ 95% CI] for POI vs. 24.8 kg/m^2^ [24.1 kg/m^2^–25.5 kg/m^2^ 95% CI] for non-POI, *p* = 0.01).

Intraoperatively, patients with POI had a significantly longer procedure duration than those without POI (313 min for patients with POI vs. 279 min for patients without POI, *p* = 0.016) (Table 4). Statistical differences in POI rates were not observed between patients with different anesthesia adjuncts (epidural or a regional block), operative approaches (laparoscopic, robotic, or open), or ileostomy type (end vs. loop) on bivariate analysis. There was a weak correlation (Schober et al., 2018) between procedure duration and POD1/POD2 fluid balance (*r* = 0.23; *p* < 0.001).

**Table 4:** Intraoperative and postoperative characteristics of study population

	All	No POI	POI	% POI	*p*-value
**Intraoperative characteristics**					
**Procedure duration (min)**	289.9	278.8	312.8	–	0.016
Anesthesia adjuncts					
Epidural	99 (37.9%)	64	35	35.4%	0.50
No epidural	162 (62.1%)	112	50	30.9%	
Operative approach					
Laparoscopic	150 (57.5%)	107	43	28.7%	0.27
Robotic	66 (25.3%)	40	26	35.6%	
Open	45 (17.2%)	29	16	39.4%	
Ileostomy type					
Diverting loop ileostomy	173 (66.3%)	112	61	35.3%	0.21
End ileostomy	88 (33.7%)	64	24	27.3%	
**Postoperative characteristics**					
Postop day 2 net fluid balance (l)	2.07	1.80	2.65	–	0.004
Postop day 2 net fluid balance					
< 807 ml	76 (29.1%)	61	15	19.7%	0.006
≥ 807 ml	185 (70.9%)	115	70	37.8%	
Postop opioid use					
Yes	223 (85.4%)	150	73	31.3%	> 0.99
No	38 (14.6%)	26	12	31.6%	

*p*-values from Fisher’s exact tests for categorical variables and *t*-tests for continuous variables

*POI* postoperative ileus

Postoperatively, patients with a POI had a lower ileostomy output on POD1 (50 mL vs. 127 mL; *p* < 0.001), lower ileostomy output on the morning of POD2 (574 mL vs. 879 mL; *p* < 0.001), and a slower diet advancement to solid food (3.6 days vs. 1.6 days; *p* < 0.001) as expected.

Patients with POI had a significantly higher net postoperative fluid balance on the morning of POD2 than those without POI in our bivariate analysis (+2.65 L vs. +1.80 L; *p* = 0.004). Those patients with fluid balance greater than + 807mL (determined as the maximum Youden index for sensitivity over 80%) were associated with a higher rate of POI (*p* = 0.006). No difference in postoperative opioid use was observed in the two groups.

In our multivariable regression analysis adjusting for age, gender, BMI, preoperative opioid use, procedure duration, and operative approach, differences in POD2 fluid balance between patients with and without POI remained significant (Table 5). In the adjusted model, for each 100-mL increase in fluid balance on the morning of POD2, the odds of POI increased by about 1.4% (*p* = 0.034). Compared to patients with a POD2 fluid balance less than + 807 mL, those with POD2 fluid balance equal to or greater than + 807 mL had 2.3 times higher odds of POI (*p* = 0.01). When adjusted for age, gender, preoperative opioid use, procedure duration, operative approach, and POD2 fluid balance, differences in BMI between patients with and without POI remained significant. No other variables, including procedure duration, showed a significant difference on multivariable modeling.

**Table 5:** Multivariable logistic regression model

	OR	95% CI		***p***-value
		Lower	Upper	
**BMI [continuous]**	**1.06**	**1.00**	**1.12**	**0.05**
Age [continuous]	1.01	0.99	1.03	0.37
Gender [male vs female]	1.39	0.77	2.49	0.27
Procedure duration [continuous]	1.00	1.00	1.00	0.64
**POD2 fluid balance [continuous]**	**1.014**	**1.001**	**1.028**	**0.034**
**POD2 fluid balance [≥ 807 vs < 807 mL]**	**2.32**	**1.19**	**4.53**	**0.01**
Preoperative opioid use [yes vs no]	1.55	0.81	2.96	0.19
Operative approach [open vs lap]	1.32	0.60	2.90	0.50
Operative approach [robotic vs lap]	1.15	0.53	2.48	0.73

*POI* postoperative ileus, *POD2* postoperative day 2

Patients in our population with POI had a significantly longer LOS (11.40 days vs. 5.12 days; *p* < 0.001) and direct cost of hospitalization ($38K vs. $22K; *p* < 0.001), with a trend toward a statistically significant difference in 30-day readmissions (24% vs. 15%, *p* = 0.086) (Table 6).

**Table 6:** Summary of outcomes associated with postoperative ileus

	All	No POI	POI	% POI	*p*-value
**Outcome measures**					
Length of stay (days)	7.16	5.12	11.40	–	< 0.001
Cost of hospitalization ($000s)	27.2	21.9	38.3	–	< 0.001
30-day readmissions					
Yes	46 (17.6%)	26	20	43.5%	0.086
No	215 (82.4%)	150	65	30.2%	

*POI* postoperative ileus

## Discussion

We examined the demographic, clinical, intraoperative, and postoperative characteristics associated with the development of POI after elective or urgent abdominal colorectal surgery that included the formation of an ileostomy as we felt that there was a gap in the literature with poor knowledge of intervenable factors that could improve clinical care in this population. We excluded emergent cases given the confounding factors, such as ongoing sepsis, bowel obstruction, poor nutrition, or need for resuscitation, that would influence POI in these patients. Understanding factors associated with POI for patients undergoing elective (non-urgent or urgent) ileostomy formation is critical to continuing to refine ERAS protocols to decrease patient discomfort, LOS, and cost of care.

The most important statistically significant, modifiable finding from our study is that a POD2 net fluid balance of greater than 800mL was associated with a higher odds of POI. We chose this time period to account for the variable intravenous fluid given to patients in the intraoperative and immediate postoperative settings. This is complicated by the fact that most patients get a full mechanical bowel prep and may come in for surgery in varying degrees of hydration. However, the period from POD0 to the morning of POD2 would allow for timely intervention. While the notion that postoperative fluid balance plays a role in the development of POI is well-supported by existing literature (Gupta & Gan, 2016) and the delay in return the return to normal bowel function has been specifically seen with perioperative fluid overload and resultant intestinal edema (Chowdhury & Lobo, 2011), the focus of the present study is limited to patients with ileostomy formation and our findings provide a specific actionable fluid balance target (+ 800 mL) for this population of patients. This is particularly salient in patients with ileostomy formation given, in addition to functional ileus, the possibility of mechanical outlet obstruction in the setting of the abdominal wall passage, a characteristic not present in other colon and rectal procedures. While it is impossible to distinguish functional ileus from mechanical obstruction given identical presenting symptoms, our findings show promise that minimizing bowel edema by ensuring POD2 fluids are less than 800 mL, and thus, avoiding lumen narrowing may have a role in minimizing POI.

Our findings support a preliminary guideline for perioperative manipulation of fluid management in patients undergoing ileostomy formation to reduce POI. Intraoperatively, this may include standardization of practices around basic fluid infusion rates, medication administration (e.g., minimization of dilution), and vasopressor vs. fluid administration in the setting of hypotension. Although dynamic measures to assess fluid responsiveness, such as pulse pressure variation, have been advocated as a guide to intraoperative fluid administration, their impact on outcomes in colorectal surgery has not been proven (McEvoy et al., 2020). Whether dynamic measures can be employed in a manner that guides clinicians toward more judicious fluid administration, in a way that leads to improved gastrointestinal outcomes (e.g., POI) in this patient population, remains to be an opportunity for future study. Given our findings showing adverse impact of positive fluid balance > 800 mL on POD 2, the role of cautious postoperative diuresis may warrant further study in circumstances in which postoperative kidney injury is not suspected. However, the only prospective study evaluating early diuresis after colon and rectal surgery to our knowledge showed negative results (Danelich et al., 2018), although that study was not limited to patients undergoing ileostomy formation. This raises the opportunity for a more focused future study of a diuresis-based intervention in the ileostomy population. Irrespective of when perioperatively the intervention takes place, a POD2 fluid balance of less than 800 mL represents an important advancement at a time when best practice targets are not defined (Chowdhury & Lobo, 2011).

Our results also show that higher patient BMI is associated with an increased rate of POI, and this remained significant on multivariable analysis. Though the body of evidence has been mixed (Danelich et al., 2018), our findings are consistent with numerous studies of patients undergoing gastrointestinal surgery (Rybakov et al., 2018; Morimoto et al., 2019) and suggest that this association may be similarly seen specifically for patients undergoing ileostomy formation. One potential explanation for this finding is the increased incidence of POI in individuals with higher BMI may indicate a mechanical effect as a result of a thicker or more muscular abdominal wall. However, this will need to be studied in further detail to confirm. Additionally, knowing that elevated BMI is associated with increased odds of POI for these patients opens the door for additional counseling regarding diet and exercise and provides opportunity for prehabilitation prior to surgery and anticipatory guidance so that patients at higher risk of POI can be best informed about what to expect during their hospitalization.

Finally, our study showed that a longer duration of the procedure was also associated with an increased rate of POI, a finding similarly supported by existing literature (Moghadamyeghaneh et al., 2016), but this association was no longer seen in our population when adjusting for BMI and fluid balance. The lack of a strong correlation between procedure duration and POD1/POD2 fluid balance suggests that case duration may be a confounding variable, and that fluid administration, as opposed to case duration, is independently associated with POI. Furthermore, given that ileostomy formation is typically part of a large operation, such as a low anterior resection or proctocolectomy with ileal pouch creation (Table 3), the length of the procedure is unlikely to be an indication of difficulty of ostomy creation but rather reflects a multitude of other components of the case. Since procedure length was not significantly associated with POI in our multivariable modeling, we can conclude that these other components of the case are not independently associated with increased POI.

While having an epidural catheter with local anesthetic has been previously shown to accelerate the return of gastrointestinal transit after abdominal surgery given its opioid-sparing properties (Guay et al., 2016), our study did not show a difference in POI between patients who received vs. those who did not receive an epidural. One potential reason may be that these studies were not limited to patients with ileostomy formation. However, another reason may be the lack of actual opioid sparing in our epidural population. The standard epidural solution compounded at our institution at the time of this study contained 2 μg/mL fentanyl in dilute (0.0625–0.1%) ropivacaine, which by itself creates significant opioid exposure over the course of its continuous administration during and after surgery. Adjusting epidural concentration or delivered analgesic may represent further opportunity for intervention.

As expected, patients in our study group with POI had lower ileostomy output on the mornings of POD1 and POD2 and were slower to advance their diet to consuming solid foods. These represent characteristic signs and symptoms of POI and are expected associations in patients with this condition. Moreover, consistent with POI in other patient groups (Peters et al., 2020), those undergoing ileostomy formation who experienced POI in our study had longer hospital stays and higher costs of hospitalization. Though not a statistically significant finding, a trend toward higher readmission rates was also seen among patients with a POI that can also contribute to the overall cost of care for these patients. These findings stress the importance of identifying ways to decrease POI as a broader measure to improve quality of care and decrease healthcare costs.

A limitation of this investigation is that it is a single-center study focused on a subset of patients (i.e., patients undergoing ileostomy formation), further limiting our patient population sample size. However, our institution is a large academic medical center reaching a sizable proportion of patients from our region. With six colorectal surgeons performing the surgeries and a 5-year study period, we were able to achieve a well-powered study and focus on a specific population of interest. Finally, there is inherent heterogeneity in the selection of patients who receive ileostomies, and differences in management given individual surgeon discretion. However, our colorectal surgery practice has several mechanisms to collaborate and share best practices, including ERAS protocols, colorectal surgery–specific order sets, and weekly service case conferences, as well as a service-specific handbook for residents, advanced care providers, and medical students to minimize variability of postoperative care. Furthermore, the number of cases here allowed us to use this variability to our advantage to hopefully generalize the results for other practices.

Despite these limitations, our study provides a preliminary guideline for intervention in patients undergoing ileostomy formation. Future research should aim to assess causality between a specific postoperative net fluid balance, such as a target of + 800 mL or less on the morning of POD2, and POI through a prospective randomized controlled trial.

## Conclusion

This study reinforces that early postoperative fluid balance plays a critical role in the prevention of POI in patients undergoing ileostomy formation, and POI in this population is associated with a longer LOS and increased cost of hospitalization. Minimizing fluid overload to prevent bowel edema, particularly in the early postoperative period, may be a potential strategy to reduce the risk of POI and thus postoperative LOS and cost of hospitalization. Fluid restriction, diuresis, and changes in diet advancement or early stoma intubation should be considered measures that may improve outcomes and should be studied more intensively.

## Data Availability

The datasets used and/or analyzed during the current study are available from the corresponding author on reasonable request.

## Abbreviations

POI: Postoperative ileus
LOS: Length of stay
ERAS: Enhanced recovery after surgery
EMR: Electronic medical record
BMI: Body mass index
POD: Postoperative day
NG: Nasogastric
DLI: Diverting loop ileostomy
